# Superior migration ability of umbilical cord-derived mesenchymal stromal cells (MSCs) toward activated lymphocytes in comparison with those of bone marrow and adipose-derived MSCs

**DOI:** 10.3389/fcell.2024.1329218

**Published:** 2024-03-11

**Authors:** Akiko Hori, Atsuko Takahashi, Yuta Miharu, Satoru Yamaguchi, Masatoshi Sugita, Takeo Mukai, Fumitaka Nagamura, Tokiko Nagamura-Inoue

**Affiliations:** ^1^ Department of Cell Processing and Transfusion, Research Hospital, The Institute of Medical Science, The University of Tokyo, Tokyo, Japan; ^2^ IMSUT CORD, Research Hospital, The Institute of Medical Science, The University of Tokyo, Tokyo, Japan; ^3^ Division of Somatic Stem Cell Research, Center for Stem Cell Biology and Regenerative Medicine, The Institute of Medical Science, The University of Tokyo, Tokyo, Japan; ^4^ Department of Obstetrics, Yamaguchi Hospital, Chiba, Japan; ^5^ Department of Obstetrics, NTT Medical Center Tokyo Hospital, Tokyo, Japan; ^6^ Division of Advanced Medicine Promotion, The Advanced Clinical Center, The Institute of Medical Science, The University of Tokyo, Tokyo, Japan

**Keywords:** mesenchymal stromal cells, umbilical cord, migration, mixed lymphocyte reaction, bone marrow, adipose tissue, chemokines, cytokine

## Abstract

**Introduction:** Mesenchymal stromal cells (MSCs) are activated upon inflammation and/or tissue damage and migrate to suppress inflammation and repair tissues. Migration is the first important step for MSCs to become functional; however, the migration potency of umbilical cord-derived MSCs (UC-MSCs) remains poorly understood. Thus, we aimed to assess the migration potency of UC-MSCs in comparison with those of bone marrow-derived MSCs (BM-MSCs) and adipose tissue-derived MSCs (AD-MSCs) and investigate the influence of chemotactic factors on the migration of these cells.

**Methods:** We compared the migration potencies of UC-, BM-, and AD-MSCs toward allogeneic stimulated mononuclear cells (MNCs) in mixed lymphocyte reaction (MLR). The number of MSCs in the upper chamber that migrated toward the MLR in the lower chamber was counted using transwell migration assay.

**Results and discussion:** UC-MSCs showed significantly faster and higher proliferation potencies and higher migration potency toward unstimulated MNCs and MLR than BM- and AD-MSCs, although the migration potencies of the three types of MSCs were comparable when cultured in the presence of fetal bovine serum. The amounts of CCL2, CCL7, and CXCL2 in the supernatants were significantly higher in UC-MSCs co-cultured with MLR than in MLR alone and in BM- and AD-MSCs co-cultured with MLR, although they did not induce the autologous migration of UC-MSCs. The amount of CCL8 was higher in BM- and AD-MSCs than in UC-MSCs, and the amount of IP-10 was higher in AD-MSCs co-cultured with MLR than in UC- and BM-MSCs. The migration of UC-MSCs toward the MLR was partially attenuated by platelet-derived growth factor, insulin-like growth factor 1, and matrix metalloproteinase inhibitors in a dose-dependent manner. Conclusion: UC-MSCs showed faster proliferation and higher migration potency toward activated or non-activated lymphocytes than BM- and AD-MSCs. The functional chemotactic factors may vary among MSCs derived from different tissue sources, although the roles of specific chemokines in the different sources of MSCs remain to be resolved.

## 1 Introduction

Mesenchymal stromal cells (MSCs) can be obtained from several sources, including the bone marrow (BM), adipose tissue (AD), and umbilical cord (UC) (Gnecchi and Melo, 2009; Gruber et al., 2010). MSCs are activated upon inflammation and/or tissue damage and migrate to suppress inflammation and repair tissues. Insulin-like growth factor (IGF-1) and platelet-derived growth factor receptor (PDGF) are the most potent chemotactic factors of BM-MSCs (Ponte et al., 2007) and AD-MSCs (Baek et al., 2011). Fetal bovine serum (FBS) containing these growth factors promotes the migration potencies of BM-MSCs (Mishima and Lotz, 2008) and AD-MSCs (Baek et al., 2011), although reports about the chemotaxis of whole UC-MSCs in the presence of FBS are lacking. The migration potencies of BM-MSCs and AD-MSCs are promoted by pre-incubating with tumour necrosis factor (TNF)-α (Ponte et al., 2007; Ponte et al., 2007; Baek et al., 2011; Baek et al., 2011). The migration potency of BM-MSCs toward injured tissues has been associated with the expression of chemokines, including stromal-derived factor-1 (SDF-1) and CXCR4 (Liesveld et al., 2020). BM-MSCs attract hematopoietic stem cells (HSCs) and provide a favorable environment for hematopoiesis. Tondreau et al. demonstrated that inflammatory cytokines promote the migratory capacity of BM-MSCs according to the expression of interleukin (IL)-6, PDGF, IGF-1, and SDF-1 receptors. The production of matrix metalloproteinases (MMP1, MMP2, and MMP13) and tissue inhibitor of metalloproteinase (TIMP1/2) also promotes migration through the extracellular matrix (Tondreau et al., 2009).

BM-MSCs and UC-MSCs have been used clinically in immunotherapy and regenerative medicine to treat acute graft-versus-host disease (GVHD) after allogeneic HSC transplantation (Le Blanc and Davies, 2015; Murata et al., 2021; Nagamura-Inoue et al., 2022), COVID-19-related acute respiratory distress syndrome (Dilogo et al., 2021; Lanzoni et al., 2021), and other inflammatory diseases. The mixed lymphocyte reaction (MLR) assay, in which lymphocyte activation is induced by the co-culture of allogeneic cells such as dendritic cells, mimics acute GVHD *in vitro*. We previously demonstrated that responder T cell proliferation triggered by allogeneic dendritic cells can be efficiently inhibited by UC-derived MSCs (UC-MSCs) from a third-party donor (He et al., 2015; He et al., 2021; Kurogi et al., 2021). We also found that UC-MSCs actively migrate toward injured cells, that is, glucose-depleted SH-SY5Y human neuroblastoma cells, *in vitro* (Mukai et al., 2016). Furthermore, we demonstrated that UC-MSCs administered intravenously into an intraventricular hemorrhage mouse model become trapped in the lungs and then accumulate in the brain, although the injected UC-MSCs could not be detected in the mice after 3 weeks (Mukai et al., 2017). However, the migration potency of UC-MSCs toward inflammatory cells in response to allogeneic stimuli remains poorly understood.

Thus, we aimed to assess the migration potency of UC-MSCs toward inflammatory cells in comparison with those of BM-MSCs and AD-MSCs. This study is the first to report the superior migration ability of UC-MSCs toward inflammatory cells in comparison with those of BM-MSCs and AD-MSCs.

## 2 Materials and methods

### 2.1 Isolation and culture of MSCs

This study was approved by the Ethics Committee of the Institute of Medical Science, University of Tokyo (IMSUT) (No. 2021-108). UC-MSCs were provided by the IMSUT Hospital Cord Blood and Cord Bank (IMSUT CORD), Japan. IMSUT CORD activity was reviewed and approved by the IRB (No. 35-2). UC-MSCs were isolated from three donors using previously reported methods (Mori et al., 2015; Shimazu et al., 2015). Briefly, frozen-thawed UC tissues were minced into 2 mm fragments and subjected to an improved explant culture procedure. Tissue fragments were placed in complete α-minimal essential medium (αMEM; Wako Pure Chemical Industries, Ltd., Japan) supplemented with 10% FBS (SERANA, Germany) and antibiotics–antimycotics (Antibiotic-Antimycotic, 100X; Life Technologies, United States) at 37°C with 5% CO_2_. Cells migrating from the UC tissue fragments were harvested using TrypLE Select (Life Technologies) and denoted as passage 1 (P1) UC-MSCs. P1 cells were frozen in StemCell Banker (Zenogen pharma Co., Ltd., Japan) (Mori et al., 2015). The 2.5 × 10^5^ frozen-thawed cells suspended in culture medium were seeded in a 10 cm culture dish and further expanded until 80%–90% confluency and passaged every 5 days with the medium refreshed every 2 days. P4 cells were used in subsequent experiments. UC-MSCs were cryopreserved in StemCell Banker and thawed before use.

Human BM mononuclear cells (BM-MNCs) (Lonza, United States) and human AD-MSCs (Lonza, United States) were purchased from LONZA KK. BM-MNCs were cultured in αMEM supplemented with 10% FBS, and the initial cells obtained were denoted as P1 BM-MSCs. BM-MSCs and AD-MSCs were cultured until P4 and used for further experiments.

### 2.2 Cell proliferation assay

The proliferation abilities of P2 UC-, BM-, and AD-MSCs were compared in αMEM supplemented with 10% FBS. In brief, 2.5 × 10^5^ cells were suspended in complete medium and plated in a 10 cm-diameter dish (n = 3 in each MSC type). The number of cells was counted using trypan blue staining under a microscope. The cumulative population doubling level (PDL) was then calculated. The PDLs of the cells at each passage were calculated using the formula 2n = Nx/N0, where Nx is the cell number after culture and N0 is the cell number before culture (Kurogi et al., 2021).

### 2.3 Analysis of surface markers in MSCs

Flow cytometry was performed as described previously (Kurogi et al., 2021; Nagamura-Inoue et al., 2022). The cells were labeled with monoclonal antibodies, which are listed in Supplementary Table S1. The cells were acquired using BD™FACSCanto II flow cytometer (BD) and analyzed using FlowJo software (BD).

### 2.4 Adipogenic, osteogenic, and chondrogenic differentiation assays

The MSCs were plated at a density of 5 × 10^4^ cells/well in 12-well plates and induced to differentiate into adipocytes with culture medium supplemented with 100 µM indomethacin (Sigma-Aldrich Co. LLC, United States), 1 µM dexamethasone (FUJIFILM Wako Pure Chemical Corporation, Japan), 0.5 µM IBMX (Sigma-Aldrich), and 10 μg/mL insulin (Sigma-Aldrich) for 2 weeks. Then, the cells were stained with Oil Red O (Sigma-Aldrich) (He et al., 2014). UC-MSCs were cultured for 4 weeks using a StemPro osteogenesis differentiation kit (Thermo Fisher Scientific Inc., United States) in accordance with the manufacturer’s instructions, and their osteogenic differentiation was evaluated. The cells were stained with alizarin red (Sigma-Aldrich). In the chondrogenic differentiation assay, we used a pellet culture system using Stem MACS™ Chondro Diff Media (Miltenyi Biotec GmbH; Germany) at 2.5 × 10^5^ in 15 mL conical tubes for 3 weeks. The cells were fixed with 4% formaldehyde and stained with toluidine blue (Sigma-Aldrich).

### 2.5 MLR

An allogeneic MLR assay was conducted as previously described (He et al., 2015; Kurogi et al., 2021; Nagamura-Inoue et al., 2022). Peripheral blood MNCs were used as the responder (R). PMDC05 cells were provided by Dr. Narita at the Faculty of Medicine, Niigata University (Narita et al., 2008; Narita et al., 2009). PMDC05 cells were irradiated and used as the stimulator (S). On the day of the MLR, R (4 × 10^5^) and S (4 × 10^4^) cells were mixed in 24-well plates at an R:S ratio of 10:1 in the presence of 0.625 ng/mL anti-human CD3 antibody (Lymactin-T; Cell Science & Technology Inc., Japan) (Nagamura-Inoue et al., 2022). The inhibition of the allogeneic MLR by MSC co-culture for 4 days in the presence of 10% FBS was investigated as previously described (Nagamura-Inoue et al., 2022) (Supplementary Figure S1).

### 2.6 Migration assays

The migratory abilities of MSCs were evaluated using a 24-well transwell chamber (Corning, United States) inserted with an 8 μm filter membrane. On the day of the migration assay, the MLR ratio described above was set in the lower chamber, and MSCs were plated at 5 × 10^3^ cells/well in the upper transwell chamber and co-cultured at 37°C with 5% CO_2_ overnight. The MSCs migrated toward the opposite side of the transwell chamber in response to the stimuli, including αMEM with 10% FBS (Montemurro et al., 2011), 4 × 10^4^ MNCs with or without MLR, primed MNCs with reagents in the lower chamber of 24-well plates, and other indicated reagents described below. Appropriate MNC number was assessed by different concentrations of cells (from 4 × 10 to 4 × 10^5^/lower chamber). Then, MSCs at the opposite filter side of the upper transwell chamber were fixed with 10% paraformaldehyde for 10 min, washed once with phosphate-buffered saline (Nissui Pharmaceutical Co., Ltd., Japan), and stained with 1 μg/mL 4′,6-diamidino-2-phenylindole, dihydrochloride (Cellstain®-DAPI, DoJin, Japan). The number of cells trapped on the opposite filter side was counted in all fields under the fluorescent microscope (×200; Niko Ti-S30-EDF-Ph-S, Nikon, Japan).

To evaluate the migration potency in response to chemokines, 1 ng/mL CCL2 (recombinant human MCP-1, Fujifilm-Wako chemicals Cor., Japan), 100 ng/mL CCL7 (Human CHO-expressed MSCP-3/CCL7, Genscript, United States), and 1 ng/mL CXCL2 (human CXCL2 protein, Acro) were added in the lower chamber of the migration assay system, after dose-dependency experiments (Data not shown).

To assess the inflammatory MNCs on the migration of UC-MSCs, MNCs primed with 10 μg/mL phytohemagglutinin-L (PHA-L, Roche, Germany), 1 μg/mL lipopolysaccharide (LPS, Fujifilm-Wako Chemicals Cor., Japan), and 10 ng/mL recombinant human TNF-α (Peprotech, United States) were incubated overnight, washed once, and then transferred to the new lower chamber of the migration assay system. The migrating cells were counted on the next day (17 h incubation). Then, the UC-MSCs were plated in the upper chamber and cultured overnight followed by counting the migrating cells as described above.

Various inhibitory factors were added to the migration assay to identify which of them induce migration. These factors included AG1296 for platelet-derived growth factor receptor (Cayman Chemical Company, United States) (Fiedler et al., 2004; Nazari et al., 2016), picropodophyllin (PPP) for insulin-like growth factor 1 receptor (IGF-1R; Merck, Deutschland) (Wang et al., 2019), and GM6001 for MMPs (Cayman Chemical Company) (Kasper et al., 2007). 5 × 10^3^ cells/well of MSCs were plated in the upper transwell chamber in the presence of 10% FBS with indicated concentration of AG1296 and PPP. For GM6001 inhibition assay, 5 × 10^3^ cells/well of UC-MSCs and 1.5 × 10^3^ cells/well of BM- and AD-MSCs were plated in the upper transwell chamber in the presence of 10% FBS., respectively.

Analysis of chemokines concentrations in the supernatant of allogeneic MLR co-cultured with UC-MSCs.

The concentrations of chemokines in the supernatant, including chemokine (C-C motif) ligand (CCL) 1, CCL2, CCL5, CCL7, CCL8, CCL11, CCL13, CCL18, CCL22, CXCL2, CXCL8, CXCL9, CXCL10, and SDF-1 content were measured using cytokine beads assay, a Human Proinflammatory Chemokine Panel (13-plex; BioLegend, United States) and Human proinflammatory chemokine Panel 2 (12-plex; BioLegend, United States) analyzed using LEGENDplex version 8.0 software (BioLegend, United States). Bead fluorescence readings were acquired using a FACSCanto II flow cytometer (BD) in accordance with the manufacturer’s instructions. All samples were analyzed in triplicate.

### 2.7 qRT-PCR analysis

Quantitative reverse transcription polymerase chain reaction (qRT-PCR) was carried out to determine the chemokine receptors and secretion of chemokines (Korbecki et al., 2020) and MMP of the MSCs. Total RNA was extracted from MSCs using Nucleospin RNA (Invitrogen Corp, Carlsbad, CA, United States). RT-PCR was performed using PrimeScript™ RT reagent kit (Takara, Shiga, Japan) in accordance with the manufacturer’s instructions. The PCR program was as follows: initial incubation at 95°C for 30 s, followed by 40 cycles of 95°C for 5 s and 60°C for 30 s. Melting curves were generated by monitoring the fluorescence of TB green signal from 95°C to 60°C, decreasing by 0.5°C for each cycle. The data were analyzed in the BioRad CFX96 real-time PCR system (BioRad, Japan). Primer sets are shown in Supplementary Table S2.

### 2.8 Statistical analysis

JMP 17.0.0 software (SAS Institute, Cary, NC, United States) was used for statistical analyses. One-way or two-way analysis of variance with Tukey’s multiple comparison test was conducted to compare differences between samples. Measurement data were expressed as mean ± SD, and *p* < 0.05 was considered statistically significant.

## 3 Results

### 3.1 Characteristics of UC-, BM-, and AD-MSCs

**FIGURE 1:**
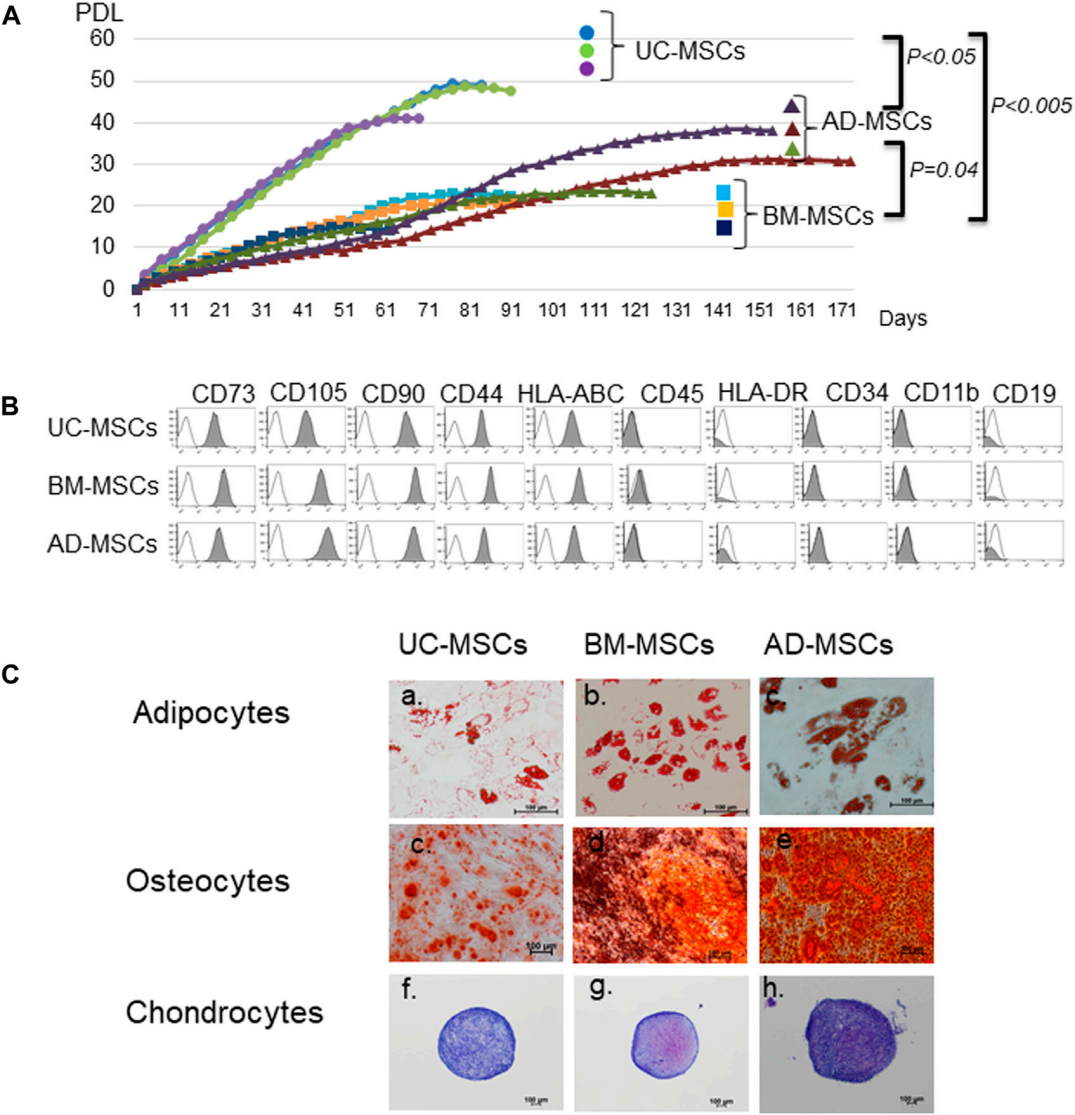
Characteristics of mesenchymal stromal cells (MSCs) from different tissue sources. **(A)** Proliferation of MSCs derived from umbilical cord (UC), bone marrow (BM), and adipose tissues (AD). Data are representative of three independent experiments and shown as mean ± SD of triplicate experiments, respectively. **(B)** Surface markers of UC-, BM-, and AD-MSCs. **(C)** Differentiation potencies of UC-, BM-, and AD-MSCs. Adipocytes are stained with oil red O, osteocytes with alizarin red, and chondrocytes with toluidine blue. UC-MSCs: umbilical cord-derived mesenchymal stromal cells, BM-MSC: bone marrow-derived MSCs, AD-MSC: adipose tissue-derived MSCs. Data are shown as mean ± SD calculated from those of three individual donors.

We compared the basic characteristics of UC-, BM, and AD-MSCs. The proliferation potencies of UC-, BM-, and AD-MSCs were compared. UC-MSCs demonstrated higher proliferation ability and speed than BM-MSCs and AD-MSCs. The proliferation limit was 46.3 ± 4.5 PDL in UC-MSCs (n = 3), 19.3 ± 4.5 in BM-MSCs (n = 3), and 31.1 ± 7.5 in AD-MSCs (n = 3; UC-MSCs vs. BM-MSCs; *p* < 0.005, UC-MSCs vs. AD-MSCs; *p* = 0.041, AD-MSCs vs. BM-MSCs; *p* < 0.05; Figure 1A). The mean ± SD of the days to reach PDL 10 was 14.8 ± 1.5 days in UC-MSCs, 28.0 ± 0 days in BM-MSCs, and 45.0 ± 11.5 days in AD-MSCs (UC-MSCs vs. BM-MSCs; *p* = 0.049*,* UC-MSCs vs. AD-MSCs; *p* < 0.005, AD-MSCs vs. BM-MSCs; P = not significant).

In accordance with the criteria of MSCs defined by the International Society of Cell & Gene Therapy (ISCT) (Dominici et al., 2006), UC-, BM-, and AD-MSCs were equally spindle-shaped, plastic-adherent cells positive for CD73, CD105, CD90, HLA-ABC, and CD44 and negative for CD45, HLA-DR, CD34, CD11b, and CD19 (Figure 1B). We also compared the abilities of UC-, BM-, and AD-MSCs to differentiate into adipocytes, osteoblasts, and chondrocytes (Figure 1C). Adipocytes stained with oil red O showed red droplets in the cells, and osteocytes with calcium deposits exhibited red particles. The pellet culture system was applied to analyze chondrogenic differentiation, and elastic firm pellets were observed. Toluidine blue staining revealed extracellular matrix formation in the cells grown in chondrogenic induction medium. Metachromasia occurred less frequently in UC-MSCs than in BM-MSCs and AD-MSCs. After immunochemical staining with alizarin red for osteogenic differentiation, UC-MSCs showed fewer calcium deposits than BM-MSCs and AD-MSCs.

### 3.2 Migration ability

**FIGURE 2:**
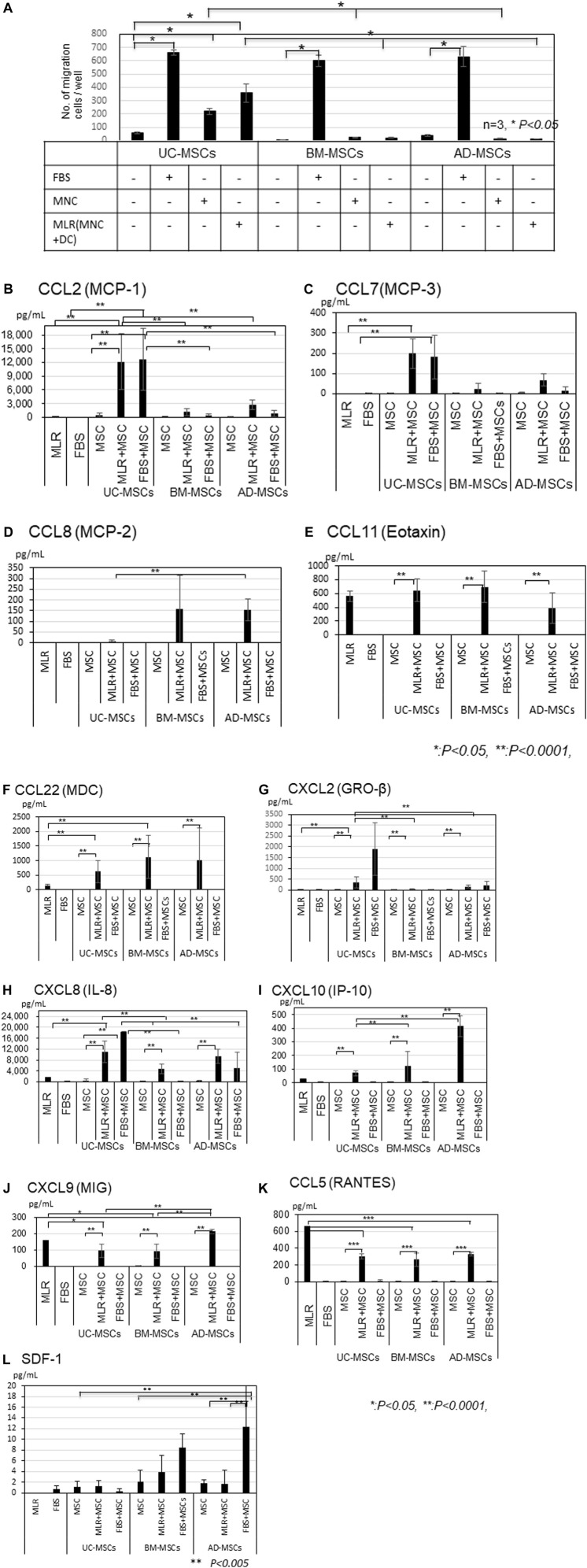
(Continued).

We compared the migration abilities of UC-, BM-, and AD-MSCs toward allogeneic MLR or FBS by using transwell migration assays (Figure 2A). FBS significantly induced migration compared with no FBS (control) in all MSCs (*p* < 0.05). The number of migrating UC-MSCs significantly increased in response to unstimulated MNCs or allogeneic stimulated MNC (MLR; *p* < 0.05), whereas the number of migrating BM- and AD-MSCs did not increase in response to both of them. UC-MSCs showed a significantly higher migration ability toward unstimulated or allogeneic stimulated MNCs than BM- and AD-MSCs (*p* < 0.05). Furthermore, the number of migrating UC-MSCs co-cultured with allogeneic MLR tended to be greater than that of unstimulated MNCs. Direct or indirect co-culture of UC-, BM-, and AD-MSCs showed no significantly different inhibitory effects on allogeneic MLR, although MLR assay was conducted in the medium supplemented with FBS (Supplementary Figure S1).

### 3.3 Chemokines in the supernatant of migration assay

CCL2, CCL7, CCL22, IL-8, and IP-10 amounts were higher in the co-cultures of UC-, BM-, and AD-MSCs with MLR than in MLR alone (Figure 2B, C, F, H, I). CCL2 and CCL7 levels were significantly higher in the supernatant of UC-MSCs co-cultured with MLR or in the presence of FBS than in that of BM- and AD-MSCs (Figure 2B, C, *p* < 0.0001), whereas CCL8 level was higher in BM- and AD-MSCs than in UC-MSCs (Figure 2D). The amounts of IL-8 were also significantly higher in UC-MSCs co-cultured with MLR than in BM-MSCs, but the difference between UC-MSCs and AD-MSCs was not significant (MLR + UC-MSCs vs. MLR + BM-MSCs; *p* < 0.05, MLR + UC-MSCs vs. MLR + AD-MSCs; not significant; Figure 2H).

IP-10 levels were induced by the co-culture with MLR and significantly higher in AD-MSCs co-cultured with MLR than in BM- and UC-MSCs (Figure 2I). CCL11, CXCL9 and CCL5 levels increased in MLR, but co-culture with MSCs did not further increase these levels (Figure 2E, J, K). Meanwhile, CCL1, CCL13, and CCL18 levels were not elevated in any type of MSCs (Supplementary Figure S2A–C). A small amount of SDF-1 was induced in the culture of BM-MSCs and AD-MSCs with FBS but not in that of UC-MSCs (Figure 2L).

### 3.4 Influence of chemokines and inflammations on the migration of MSCs

To determine whether the chemokines elevated in the supernatant of UC-MSCs co-cultured with MLR can increase the migration potency of UC-MSCs in an autocrine manner, we directly added CCL2 (n = 3), CCL7 (n = 3), and CXCL2 (n = 3) in the MSCs and evaluated the induction of migration. Even when large amounts of CCL2, CCL7, and CXCL2 were secreted by UC-MSCs co-cultured with MLR, they did not increase the migration potency of UC-MSCs. CCL2, CCL7, and CXCL2 also did not increase the migration potencies of BM- and AD-MSC. The major CCL2 receptor, CCR2 was not expressed on UC-, BM-, and AD-MSCs. CCL2 receptors (CCR1, CCR2, CCR3, CCR4, and CCR5) and CCL7 receptors (CCR1, CCR2, CCR3, and CCR5) cross interfere for several ligands. Quantitative qRT-PCR and flow cytometry analysis results showed that UC-, BM, and AD-MSCs tested weak positive for CCR1 and less weak for CCR4 but not for CCR2, CCR3, and CCR5 with or without 10% FBS supplementation (Supplementary Figure S4A–E). qRT-PCR data also showed that the CXCL2 receptor CXCR2 was not expressed at all in the MSCs (Supplementary Figure S4F).

**FIGURE 3:**
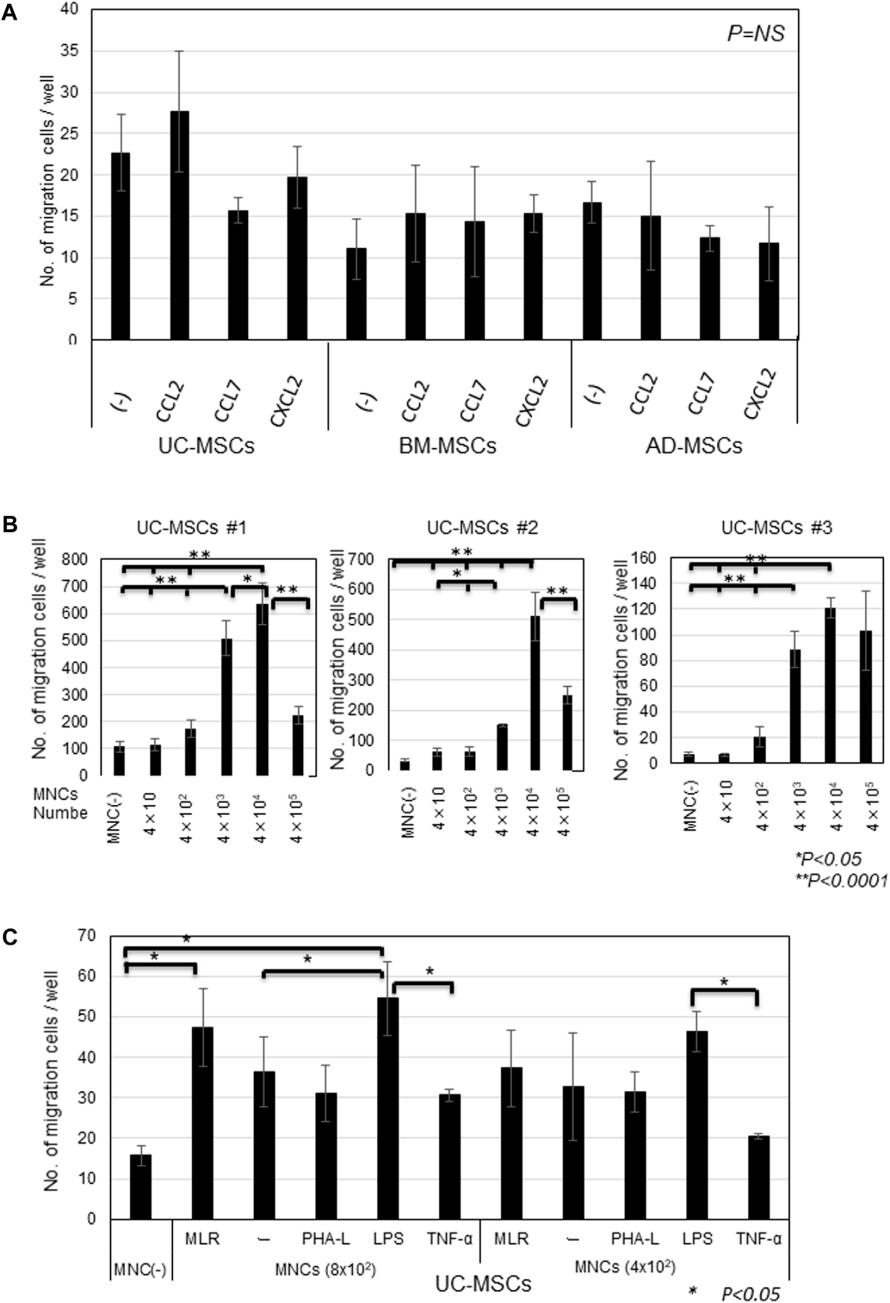
Migration of MSCs in response to CCL2, CCL7, and CXCL2 and MNCs stimulated by inflammatory factors **(A)** Migrated cell counts of UC-, BM-, and AD-MSCs in response to CCL2, CCL7, and CXCL2. 1 ng/mL CCL2, 100 ng/mL CCL7, and 1 ng/mL CXCL2 (human CXCL2 protein, Acro) were added in the lower chamber of the migration assay system. Representative data are shown as MSCs from three donors, respectively. **(B)** Migrating cell counts of UC-MSCs toward different doses of MNCs. Data are shown as three individual experiments of UC-MSCs derived from three donors. *p < 0.05, **p < 0.0001. **(C)** Migrated cell counts of UC-MSCS in response to MLR and MNCs primed with PHA-L, LPS, and TNF-α, respectively. Different MNC numbers in the lower chambers before stimulation are shown. Representative data of three independent experiments with mean ± SD are shown. *p < 0.05.

Considering that UC-MSCs seemed sensitive to migrate toward MNCs, we first assessed the influence of MNC dose on the migration potency of UC-MSCs to identify whether the inflammatory MNCs can increase the migration potency of UC-MSCs. UC-MSCs migrated toward MNCs in a dose-dependent manner at the range of 4 × 10–4 × 10^4^ in the lower chamber of 24-well plates; however, interestingly, the excess confluency at 4 × 10^5^ suppressed the migration (Figure 3B).

Thus, we used the lower dose of MNCs for proliferation in the chamber. We primed MNCs with inflammatory reagents, such as PHA-L, LPS, and TNF-α. MNCs primed with LPS increased the migration of UC-MSCs compared with MNCs alone at 8 × 10^2^ MNCs/well, but the influence of LPS-primed MNCs on the migration was observed less at the lower amount of MNCs (4 × 10^2^; Figure 3C). Conversely, the migration of UC-MSCs toward MNCs primed with PHA-L and TNF-α was attenuated rather than accelerated.

### 3.5 Influence of inhibitors on the migration of MSCs

**FIGURE 4:**
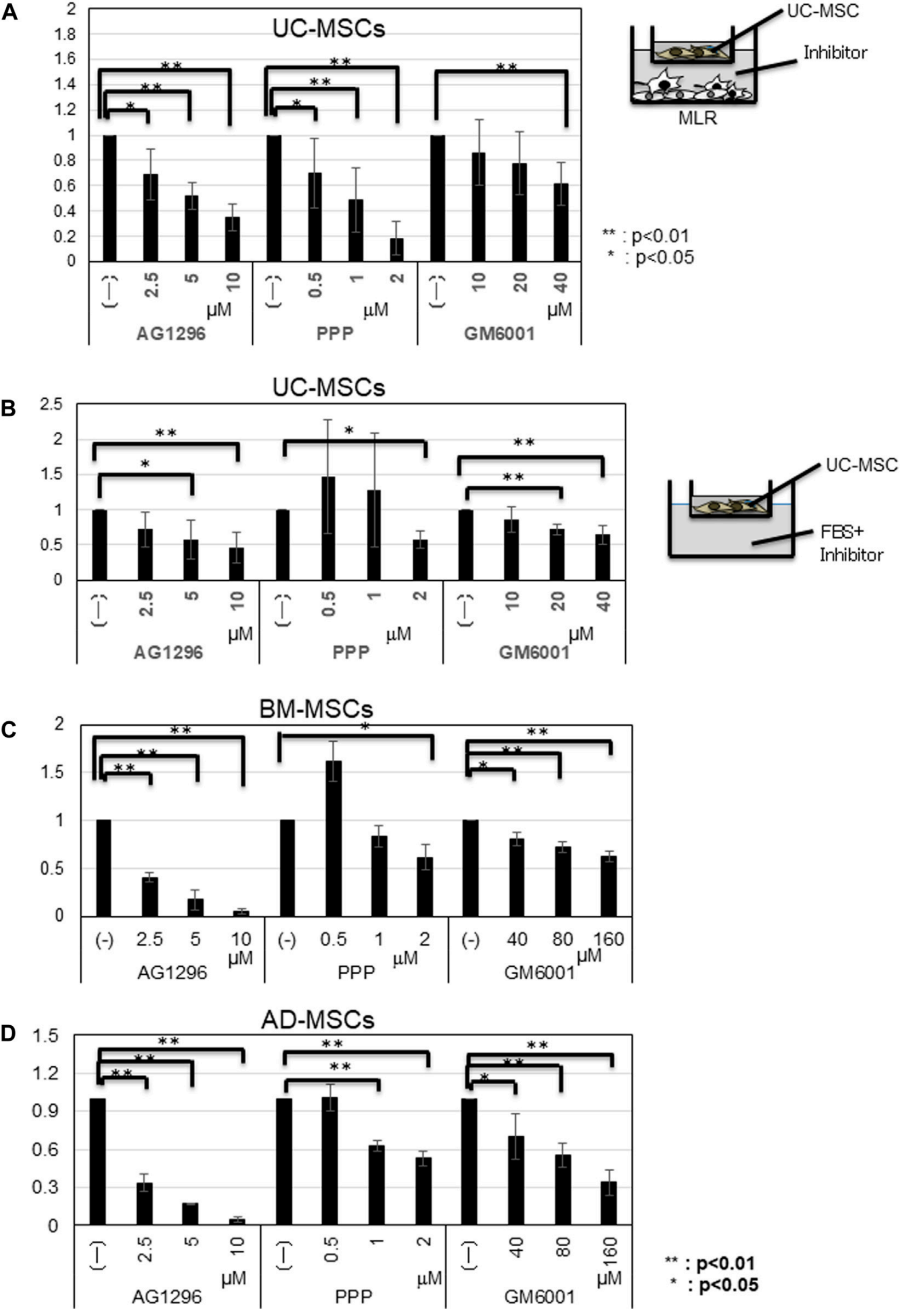
Influence of inhibitors on the migration of MSCs toward MLR. **(A)** Migrated cell counts of UC-MSCs in response to MLR with indicated inhibitors. AG1296; PDGF (platelet-derived growth factor) inhibitor; PPP; IGF-1 (insulin-like growth factor-1) inhibitor, and GM6001; MMP (matrix metalloproteinases) inhibitor. **(B)** UC-MSCs; **(C)** BM-MSCs; **(D)** AD-MSCs in the presence of FBS and inhibitors. Data are shown as mean ± SD calculated from migrated cell numbers of three individual donors, relative to the control (MSCs alone). 5 × 10^3^ cells/well of MSCs were plated in the upper transwell chamber with indicated concentration of AG1296 and PPP. For GM6001 inhibition assay, 5 × 10^3^ cells/well of UC-MSCs and 1.5 × 10^3^ cells/well of BM- and AD-MSCs were plated in the upper transwell chamber, respectively. *p < 0.05, **p < 0.01.

The amounts of CCL2, CCL7, and CXCL2 were elevated in UC-MSCs co-cultured with MLR, but these chemokines did not increase the migration potency of UC-MSCs. We then studied the influence of PDGF, IGF-1, and MMPs, which are well-known migration growth factors, on the migration of MSCs. Considering that these factors have several subtypes, we used inhibitors for PDGFA/B, IGF-1, and MMPs (MMP2, MMP9, and MMP14). We added AG1296 for PDGF, PPP for IGF-1, and GM6001 for MMPs, into the migration system of UC-MSCs toward the MLR. The migration of UC-MSCs toward the MLR was inhibited by the PDGF, IGF-1, and MMPs inhibitors in a dose-dependent manner (Figure 4A).

Supplementation with 10% FBS increased the migration potency of all types of MSCs. Thus, we conducted an inhibition assay using inhibitors for PDGFA/B, IGF-1, and MMPs. In the presence of FBS, the migration of all types of MSCs was inhibited by AG1296, PPP, and GM6001 in a dose-dependent manner. The migration of BM- and AD-MSCs required a higher GM6001 dose (Figure 4B–D). qRT-PCR results demonstrated that the amounts of MMP2, MMP9, and MMP14 increased more in BM- and AD-MSCs than those in UC-MSCs (Supplementary Figure S4C–E).

## 4 Discussion

UC-MSCs grow faster and have a higher maximal proliferation limit than BM-and AD-MSCs. Rapid proliferation increases the number of cells in a limited period and reduces the culture cost. UC-MSCs could proliferate up to more than 40 PDL, AD-MSCs approximately 30 PDL, and BM-MSCs less than approximately 20 PDL. UC, BM, and AD-MSCs expressed the same surface markers as those defined by ISCT. However, UC-MSCs showed less potency for differentiating into osteocytes than BM- and AD-MSCs, which is consistent with the finding of a previous study (Drela et al., 2016; Calcat et al., 2023). Hsieh et al. (2010) demonstrated that WJ-MSCs (UC-MSCs without vessels before culture) express more angiogenesis- and growth-related genes, including epidermal growth factor and FLT1, whereas BM-MSCs express more osteogenic genes, such as RUNX2, DLX5, and NPR3 (Hsieh et al., 2010). The gene expression pattern of BM-MSCs is more similar to that of osteoblasts than WJ-MSCs, suggesting a better osteogenic potential. By contrast, WJ-MSCs are more primitive because they share more common genes with embryonic stem cells and can less differentiate into osteocytes. We also demonstrated through PCR that UC-MSCs express Oct4, Nanog, and SSEA3/4 (He et al., 2014). Drela et al. (2016) reported that Wharton’s jelly-MSCs (UC-MSCs without vessels) exhibit a higher proliferation rate than BM-MSCs, representing an example of immature-type “pre-MSC,” which is largely composed of embryonic-like, pluripotent cells with the default neural-like differentiation.

Migration is the first important step for MSCs to become functional. Compared with BM- and AD- MSCs, UC-MSCs showed significantly higher migration potency toward unstimulated MNCs and allogenic MLR. In corresponding to higher migration, the CCL2, CCL7, and CXCL2 levels were significantly higher in UC-MSCs co-cultured with MLR than in MLR, FBS, MNCs, and MSCs alone and in BM- and AD-MSCs co-cultured with MLR. Specifically, their levels were more than 10-fold greater in MLR with UC-MSCs than in MLR with BM- and AD-MSCs, suggesting a unique response of UC-MSCs to FBS and inflammation. These chemokines were not elevated in MLR or MLR with BM-, AD-MSCs, such as CCL11, MIG, and RANTES. We hypothesized that UC-MSCs increase the migration potency in response to the self-secreted CCL2, CCL7, and CXCL2 by an autocrine mechanism. However, none of these chemokines could significantly induce the migration of UC-MSCs. To prove these results, we studied the possible receptors for CCL2, such as CCR1, CCR2, CCR3, CCR4, and CCR5 (She et al., 2022). Flow cytometry results showed negative expression of CCR2, CCR3, and CCR5; moreover, CCR1 and CCR4 had low mRNA levels in UC-MSCs (Supplementary Figure S3A–E), although the expression levels of BM- and AD-MSCs are controversial (Ponte et al., 2007; Baek et al., 2011; She et al., 2022). Ponte et al. reported BM-MSCs did not migrate towards CCL2 (MCP-1), although low-positive for CCR2, positive for CCR3, CCR4, and CCR5. UC-MSCs did not respond to these chemokines, suggesting that these chemokines may educate or recruit the other cells in response to MNC in MLR rather than induce chemotaxis of UC-MSCs.

CCL2, CCL7, and CXCL2 are potent chemotactic factors that cause the accumulation of monocytes polarized from M1 to M2 macrophages (Sierra-Filardi et al., 2014; Bao et al., 2022; Wu et al., 2022). Recently, CCL2 has attracted attention as a target in cancer therapy because of its immunosuppression in cancer extensions (Fei et al., 2021; Fei et al., 2021). Sierra-Filardi et al. (2014) (Sierra-Filardi et al., 2014) found that CCL2 determines the extent of macrophage polarization from M1 to M2, whereas CCL2-CCR2 blockade by CCR2 upregulates the expression of M1 polarization-associated genes and cytokines and downregulates the expression of M2-associated markers in human macrophages (Wu et al., 2022). CCL2 recruits not only monocytes but also regulatory T cells, neural progenitor cells, microglia, hepatic stellate cells, and several tumor cells, which express CCR2. CCL2 knockout causes abnormal monocyte recruitment in mice and several inflammatory models *in vivo* and influences the expression of cytokines related to T helper responses (Lu et al., 1998). Zhang et al. (2021) (Zhang et al., 2021) reported that human BM-MSCs enhance the chemotaxis of T cells activated by IL-2 and inhibit their proliferation through the CCL2–CCR2 axis. Furthermore, Cao et al. (2021) (Cao et al., 2021) demonstrated that CCL2 plays an important role in the treatment of idiopathic pneumonia syndrome (IPS) in an acute GVHD mouse model. In an IPS mouse model, the application of mouse BM-MSCs prolongs survival and reduces pathological damage and T cell infiltration into the lung tissue, whereas the administration of CCR2 or CCL2 antagonists in MSC-treated mice significantly attenuates the prophylactic effect of MSCs on IPS. Although these previous studies demonstrated that the CCL2-CCR2 axis is related to BM-MSCs, UC-MSCs secreting higher levels of CCL2 and CCL7 than BM-MSCs may have some advantages over BM-MSCs in treating IPS as a complication of HSC transplantation. Wu et al. (Wu et al., 2022) found through lung metastasis analysis that CCL7 is highly expressed in lung adenocarcinoma and that its knockdown suppresses chemotaxis and M2 skewing in macrophages. Bao et al. (2022) (Bao et al., 2022) reported that CXCL2 is highly expressed in the lung metastasis of colorectal cancer and induces the activation and attraction of M2 macrophages. Taken together, our results suggest that inflammation induces UC-MSCs to secrete CCL2, CCL7, and CXCL2, which may not activate the migration of UC-MSCs, but may accumulate and recruit macrophages to polarize into the M2 phenotype to control inflammation. The mechanisms and functions of these chemokines in immune systems have not been fully understood, and the effects of CCL2, CCL7, and CXCL2 secreted by UC-MSCs on the immune cells remained to be elucidated. Thus, further studies should focus on the migration of MNCs toward UC-MSCs and/or the direct influence of UC-MSCs co-cultured with MLR on the immune cells to elucidate the function of MSCs.

With regard the other cytokines in UC-MSCs, IL-8 was also significantly induced in UC-MSCs co-cultured with MLR or in the presence of FBS, compared with that in BM-MSC co-cultured with MLR and FBS. IL-8 (CXCL8) is an inflammatory cytokine that induces neutrophil chemotaxis, phagocytosis, and angiogenesis. In the MLR suppression and regulatory T cell induction, Barcia et al. (2015) (Barcia et al., 2015) reported that UC-MSCs are less immunogenic and show higher immunosuppressive activity than BM-MSCs. UC-MSCs showed lower expression levels of HLA-DR, HO-1, IGFBP1/4/6, ILR1, IL6R, and PTGES and higher expression levels of CD200, CD273, CD274, IL1B, IL-8, LIF, and TGFB2 than BM-MSCs, although the functional role of IL-8 in the suppression of MLR remains to be elucidated. By contrast, CXCL9 (MIG), CCL5 (RANTES), and CCL11 (Eotaxin) levels were elevated in MLR, and co-culture of MSCs with MLR did not increase these levels further, suggesting that these chemokines may induce the migration of MSCs or activate the functions. The levels of inflammatory cytokine CXCL10 (IP10) were higher in AD-MSCs than in UC- and BM-MSCs. Although the role of IP-10 in AD-MSCs co-cultured with MLR was not yet clarified, the potency of AD-MSCs in suppressing activated T cells in allogenic MLR was not inferior to those of UC-MSCs and BM-MSCS *in vitro*. In the present study, SDF-1 levels were higher in BM-MSCs and AD-MSCs than in UC-MSCs. This result is theoretical because BM-MSCs support HSC expansion in the BM (Marquez-Curtis and Janowska-Wieczorek, 2013), although the high SDF-1 secretion of AD-MSCs is unexpected.

We next studied the migration potency of UC-MSCs co-cultured with MNCs stimulated by PHA-L, LPS, and TNF-α. Interestingly, the migration potency of UC-MSCs toward MNCs increased when stimulated with LPS, but MNCs stimulated by PHA-L and TNF-α failed to promote the migration of UC-MSCs. We could not exclude the direct interaction of these reagents in the study, even though we washed once before co-culture. Interestingly, UC-MSCs were sensitive to migrate toward the unstimulated MNCs in a dose-dependent manner, although packed high concentration showed the reverse results, suggesting the inhibition factors secreted by packed MNCs. We could not measure and analyze the chemokines in the study. These results suggested that UC-MSCs are sensitive to migrate toward MNCs, but excess dose or excess stimuli may suppress the migration.

Taken together with the above results, we needed to identify the key factors inducing the migration of UC-MSCs co-cultured with MNCs and MLR. Previous studies reported that PDGFA/B, IGF-1, and MMPs (MMP2, MMP9, MMP14, and TIMP1/2) are the key factors inducing the migration in the presence of FBS, a secure migration inducer (Mishima et al., 2010). Considering that these factors have several subtypes, we used the inhibitors AG1296 for PDGF, PPP for IGF-1, and GM6001 for MMPs and found that the migration of UC-MSCs was partially attenuated by the addition of these inhibitory factors in a dose-dependent manner. The results suggested that PDGFA/B, IGF-1 and MMPs play important roles even in the migration of UC-MSCs toward the MLR. We also conducted inhibition assays using UC-, BM-, and AD-MSCs supplemented with FBS as the control. As expected, the migration potencies of the three types of MSCs were attenuated by PDGF, IGF-1, and MMP inhibitors. The MMP inhibitor GM6001 was required more concentration in BM-, and AD-MSCs than UC-MSCs, possibly because of the larger amount of MMPs in these MSCs than in UC-MSCs (Supplementary Figure S4). In the present study, we did not demonstrate the influence of UC-MSCs on MNC characteristics, including polarization. To clarify the benefit of UC-MSCs, we need to identify the specific factors secreted by MLR or receptors in UC-MSCs.

This study has some limitations. In the relationship of migration/the specific elevation of chemokines in UC-MSCs and immunosuppressive potency, the three types of MSCs also showed no difference in MLR inhibitory effect (Supplementary Figure S1). Migration and MLR inhibition assays are carried out under different conditions because FBS is required in the MLR inhibition assay (Nagamura-Inoue et al., 2022). Moreover, the co-culture period differs between the migration assay (overnight) and MLR inhibition assay (4 days). Therefore, we could not conclude that the migration ability is reflected in our MLR inhibition ability. Thus, time-course experiments may be required to identify additional differences among chemokines or MSCs in the future. However, the fact that UC-MSCs may be more sensitive to migrate to the activated or non-activated lymphocytes than BM- and AD-MSCs might be one of the advantages of UC-MSCs over the two other MSC types in the clinical treatment of acute GVHD.

In conclusion, UC-MSCs showed faster proliferation and higher migration potency toward activated or non-activated MNCs than BM- and AD-MSCs. The functional chemotactic factors may vary among MSCs derived from different tissue sources, although the role of specific chemokines in the different sources of MSCs remained to be resolved.

## Data Availability

The original contributions presented in the study are included in the article/Supplementary Material, further inquiries can be directed to the corresponding author.
