# Supplementary material for: Superior migration ability of umbilical cord-derived mesenchymal stromal cells (MSCs) toward activated lymphocytes in comparison with those of bone marrow and adipose-derived MSCs
Source: Front Cell Dev Biol. 2024 Mar 11;12:1329218. doi: 10.3389/fcell.2024.1329218 (PMC10961348; doi:10.3389/fcell.2024.1329218)
Supplement: Supplementary file 1 [file DataSheet1.pdf]

Supplementary Material

Supplementary Figures

Supplementary Figure 1. Inhibitory effects of co-culture of umbilical cord (UC), bone marrow (BM), and adipose tissue (AD)-derived mesenchymal stromal cells (MSCs) on allogeneic mixed lymphocyte reaction (MLR).

Black columns show the comparison of the inhibitory effect of activated T cell proliferation in MLR by direct co-culture with UC-, BM-, and AD-MSCs, whereas white columns show those by indirect co-culture with them separated by a filter chamber. Mean±SD % of inhibition of MLR stimulation by MSCs is shown (n=3 each MSCs). The inhibitory effect was calculated as previously described (1). *P=NS (not significant).*

The inhibition of allogeneic MLR by co-culture of MSCs was analyzed as previously reported (1). For MLR, mononuclear cells (MNCs) stained with 5-(and -6)-carboxyfluorescein diacetate succinimidyl ester (CFSE) (Vybrant CFDA SE Cell Tracer Kit; Invitrogen) and S cells (PMDC05 cells) were mixed and co-cultured with or without MSCs. After 4 days of culture, the CFSE fluorescence intensities of the R T cells in the CD4+ and CD8+ gates were analyzed using FACS Canto II and FlowJo software. The immunosuppressive effects of the MSCs were compared based on the blockade of daughter cell peaks. Briefly, the parental intensity of the non-proliferated CFSE histogram gate was the standard line, which is indicated as M1, and the subsequent gates (M2, M3, M3, etc.) of the proliferated daughter cells were manually set to achieve approximately two-fold progressive reductions in CFSE intensity. The inhibition ratio (%) was calculated as the ratio of each division index, R+S+MSCs and R+S (100%). In the transwell assay for MLR inhibition, we placed MLR in the upper chamber and MSCs in the lower chamber.

1. Nagamura-Inoue T, Kato S, Najima Y, Isobe M, Doki N, Yamamoto H, et al. Immunological influence of serum-free manufactured umbilical cord-derived mesenchymal stromal cells for steroid-resistant acute graft-versus-host disease. International journal of hematology. 2022;116(5):754-69.

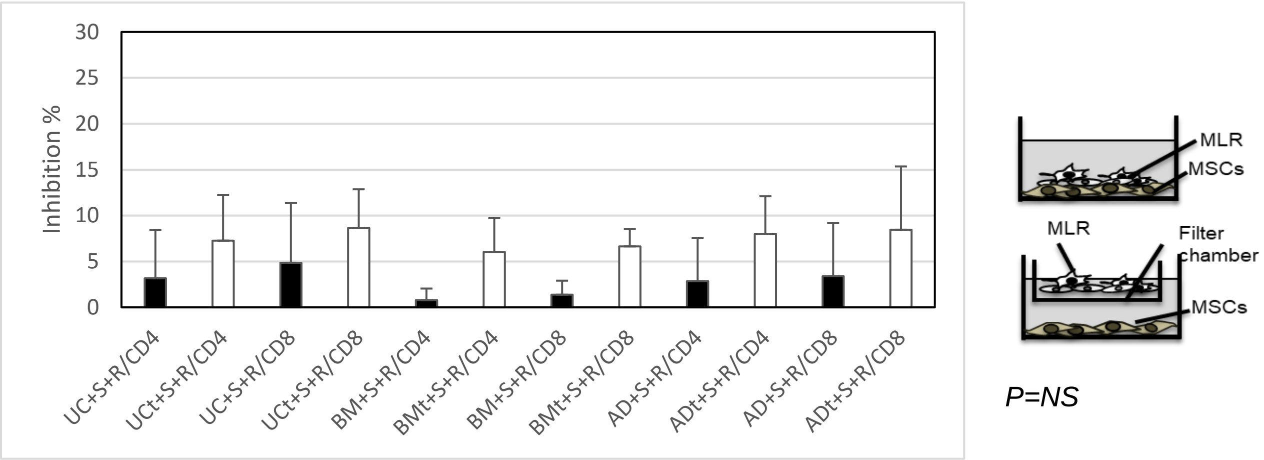

P=NS

Supplementary Figure 2. Chemokine levels in the supernatant.

The supernatant of mesenchymal stromal cells (MSCs) in the presence or absence of fetal bovine serum (FBS) and co-culture with mixed lymphocyte reaction (MLR). (**A**) chemokine (C-C motif) ligand (CCL)11 (I-309), (**B**) CCL13 (MCP4), and (**C**) CCL18 (PARC). Data are shown as mean±SD calculated from those of three individual donors.

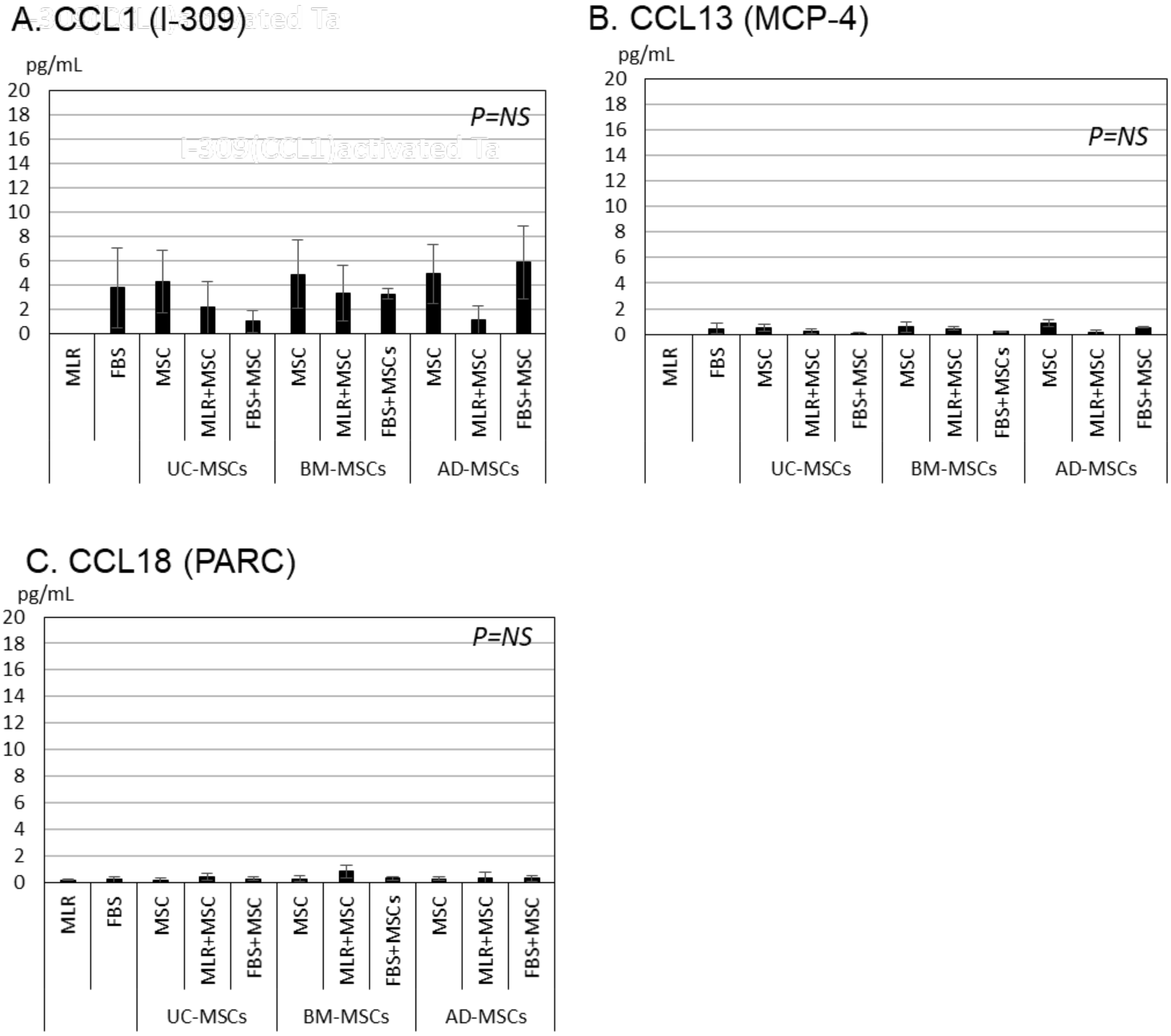

Supplementary Figure 3. Expression of chemokine receptors and chemokines in mesenchymal stromal cells (MSCs)

Gene expression levels of chemokine receptors CCR1 **(A)** and CCR4 **(B)** in umbilical cord (UC)-, bone marrow (BM)-, and adipose tissue (AD)-MSCs derived from three donors were analyzed using qRT-PCR. Data are shown as mean ± SD in triplicate. Representative data of expression of CCR2 **(C)**, CCR3 **(D)**, and CCR5 **(E)** are shown in UC-, BM-, and AD-MSCs derived from three donors. Histograms are shown gating the CD73-positive and CD1-5-positive MSCs. Positive controls of CCR2, and CCR3 using peripheral blood mononuclear cells (PB-MNCs) gated monocyte region are shown in the right lane. Positive control of CCR5 using THP-1 (Leukemia cell line). Gene expression of CXCR2 **(F)** analyzed by qRT-PCR, and the positive control is PB-MSCs. CXCR2 was not expressed in UC-, BM, and AD-MSCs. PB-MNCs gated monocyte region are shown in the left bar.

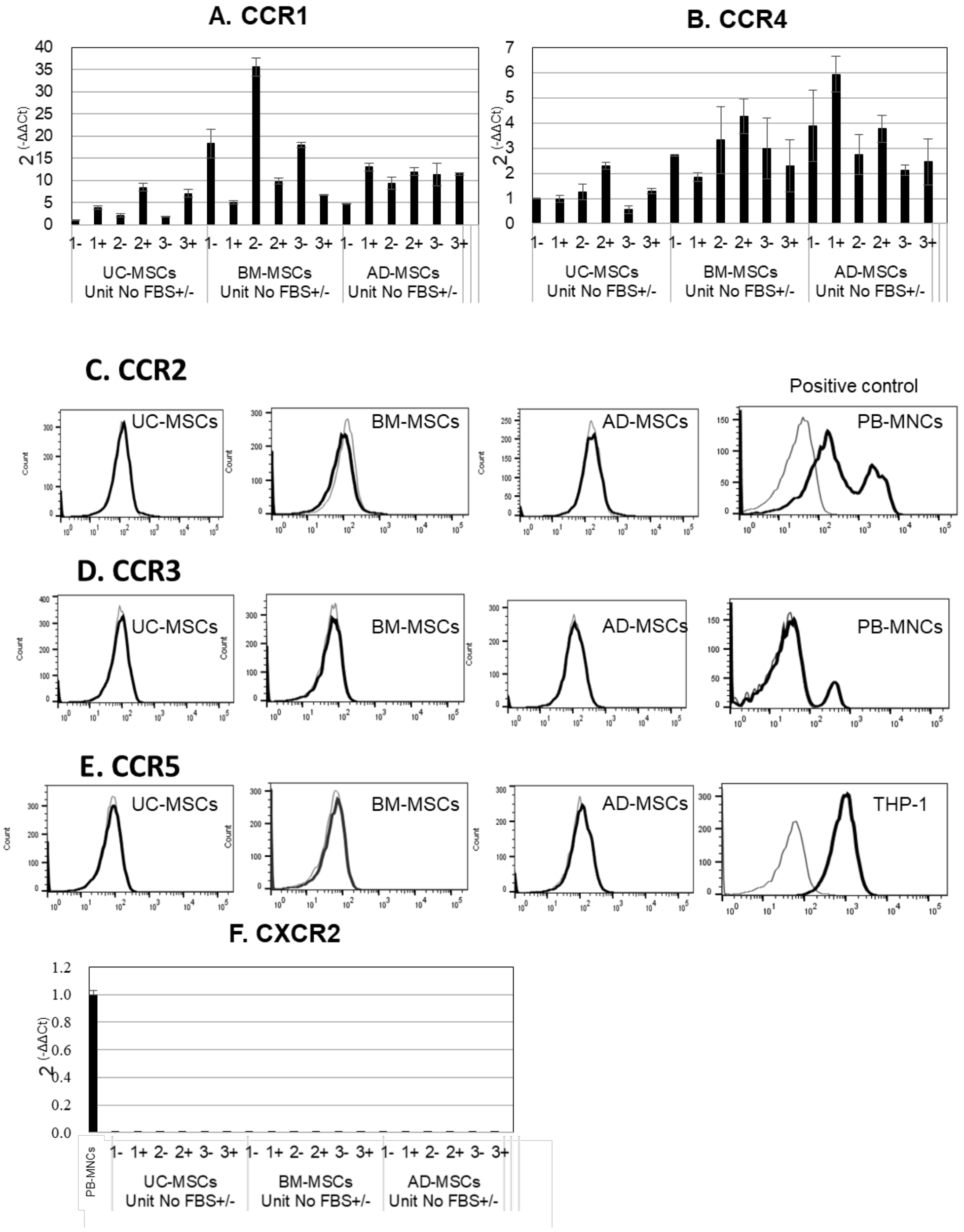

Supplementary Figure 4. Gene expression of platelet-derived growth factor receptor (PDGF), insulin-like growth factor (IGF-1), and CXCL2 receptors in MSCs

RNA expression of receptors, PDGFRα for PDGF **(A)**, IGF-1R for IGF-2 **(B)**, and matrix metalloproteinase (MMPs), including MMP2 **(C)**, MMP9 **(D)**, and MMP14 **(E),** was measured using qRT-PCR.

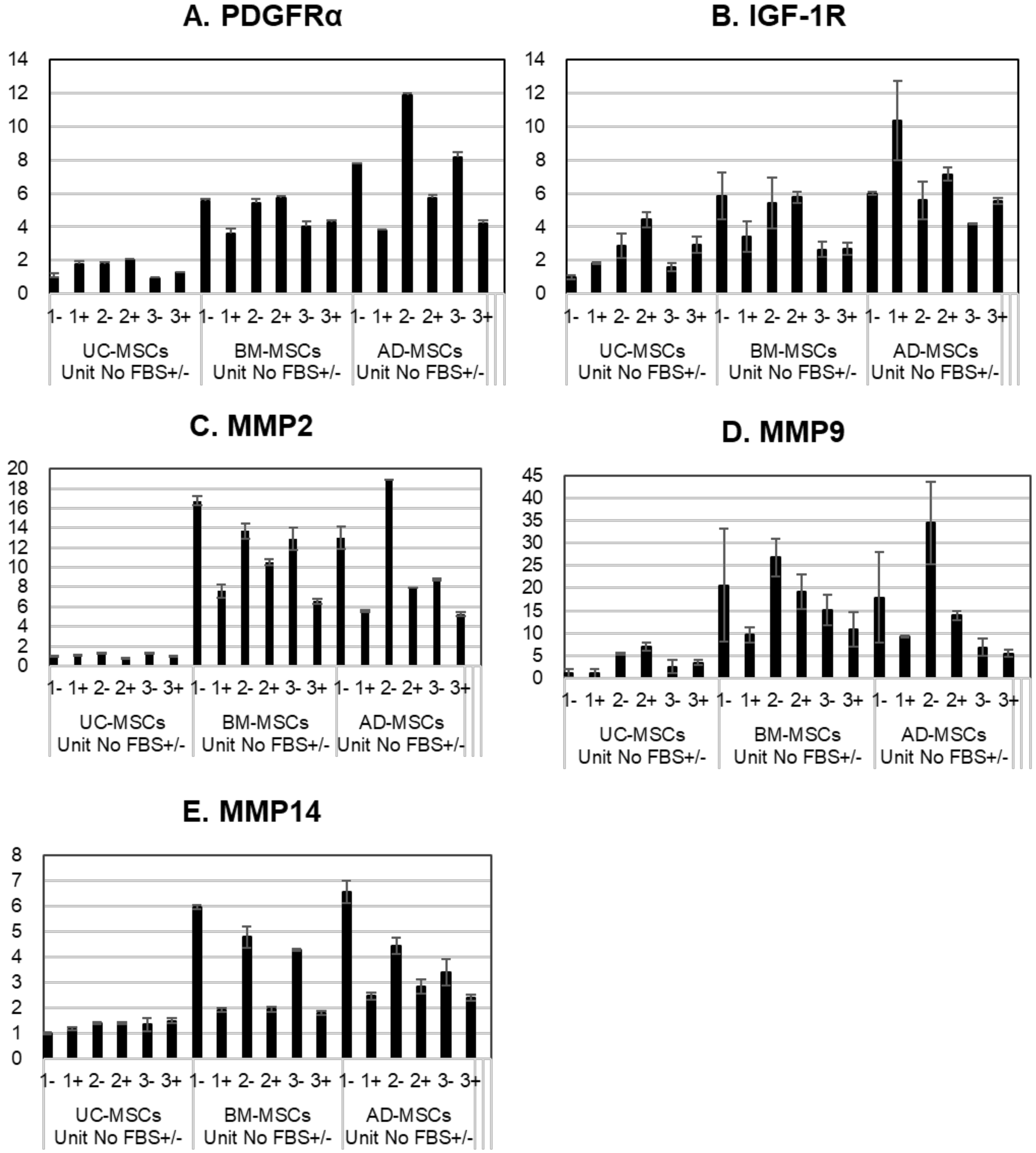

Supplementary table 1. Mouse monoclonal Antibodies and reagents used for flow cytometry

Antibodies	Company	Reference No,
Surface marker of MSCs		
FITC-conjugated -CD90	Becton & Dickinson Bioscience (BD Bioscence, USA)	555595
PerCP-anti-CD73		561260
APC-anti-CD105		562408
APC-anti-CD45		555485
FITC-anti-CD44		347943
FITC-anti-CD34		348053
PE-anti-CD73		550257
PE-anti-CD11b		561001
PE-anti-CD19		561741
FITC-anti-HLA-ABC	Beckman Coulter (BC) Inc.; (BC, USA)	IM1838U
PE-anti-HLA-DR	BC	IM0464U
IOTest IgG FITC	BC	A07795
IOTest IgG PE	BC	A07796
IOTest IgG APC	BC	IM2475
Mouse IgG PerCP Isotype	BD Bioscence	4276971
Mixed lymphocyte reactions		
7AAD	BD Bioscence	559925
PE-Cy7-anti-CD4	BD Bioscence	557852
APC-anti-CD8	BD Bioscence	340584
Chemokine receptors		
APC-anti-CCR2	BD legend	357201
FITC-anti-CCR3	Abcom	ab270646
PE-anti-CCR5	Abcom	ab176551
APC-IgG2a κ	Biolegend	400221
FITC-IgG2b	Abcom	ab91368
PE-anti-CCR5	BC	A07796

Supplementary table 2. Primer set of qRT-PCR

Primers	Sequences	Length of the product
Human CCR1-forward	GACTATGACACGACCACAGAGT	128 bp
Human CCR1-reverse	CCAACCAGGCCAATGACAAATA	
Human CCR3-forward	CTACTCCCACTGCTGCATGA	172 bp
Human CCR3-Reverse	TGCTGTGGATGGAGAGACAG	
Human CCR4-forward	AGAAGGCATCAAGGCATTTGG	137 bp
Human CCR4-reverse	ACACATCAGTCATGGACCTGAG	
Human PDGFB-forward	TGAGAAAGATCGAGATTGTGCG	121 bp
Human PDGFB-reverse	GGGCTTCGGGTCACAGG	
Human CXCR2-forward	GCAACCCAGGTCAGAAGTTTCAT	236 bp
Human CXCR2-reverse	TCAAAGCTGTCACTCTCCATGTT	
Human IGF-1R-forward-1	GCTGCTCGAGTAGGTCTTGG	119 bp
Human IGF-1R-reverse-1	ACACGCAACCCTTGACTGAT	
Human MMP2-forward	ACTACAACTTCTTCCCTCGCA	104 bp
Human MMP2-Reverse	GGCATCATCCACTGTCTCTG	
Human MMP9-forward	GTACTCGACCTGTACCAGCG	92 pb
Human MMP9-Reverse	AGAAGCCCCACTTCTTGTCG	
Human MMP14-forward	GAGCTCAGGGCAGTGGATAG	172 pb
Human MMP14-Reverse	GGTAGCCCGGTTCTACCT	
Human GAPDH-Forward	AGC CTC AAG ATC ATC AGC AAT G	111 bp
Human GAPDH-Reverse	ATG GAC TGT GGT CAT GAG TCC TT	
